# Sevoflurane promotes the apoptosis of laryngeal squamous cell carcinoma in-vitro and inhibits its malignant progression via miR-26a/FOXO1 axis

**DOI:** 10.1080/21655979.2021.1962684

**Published:** 2021-09-13

**Authors:** Dan Liu, Lang Wan, Hao Gong, Shiming Chen, Yonggang Kong, Bokui Xiao

**Affiliations:** a Department Of Otorhinolaryngology, Huangshi Central Hospital Of Edong Healthcare Group, Hubei Polytechnic University, Huangshi City, Hubei Province, China; b Department Of Anesthesiology, Huangshi Maternity And Children’s Health Hospital, Huangshi City, Hubei Province, China; c Department Of Otolaryngology Head And Neck Surgery, Renmin Hospital Of Wuhan University, Wuhan City, Hubei Province, China; d Otorhinolaryngology-Head And Neck Surgery Laboratory, Wuhan University School Of Medicine, Wuhan City, Hubei Province, China

**Keywords:** Sevoflurane, miR-26a, foxo1, laryngeal squamous cell carcinoma

## Abstract

Laryngeal squamous cell carcinoma (LSCC) is a laryngeal malignancy with a high mortality rates, and its treatment remains difficult. Sevoflurane is a surgical anesthesia which has anti-tumor effect. This investigation assessed the effects of LSCC cells treatment with Sevoflurane in vitro and in vivo. Hep-2 and Tu177 cells, human LSCC samples and BALB/C nude mice were used for result assessments. Cell viability, proliferation, migration and invasion were assessed via Cell Count Kit-8, wound healing assay and transwell invasion assay respectively. MiR-26a and FOXO1 expressions was examined by qRT-PCR. FOXO1, E-cadherin, N-cadherin and vimentin activities were examined by Western blotting. Moreover, animal experiments were performed to verify our findings *in vitro*. Lastly, miR-26a and FOXO1 expression levels in clinical samples were analyzed. According to the results, Sevoflurane decreased LSCC cells’ viability and even stimulated their apoptosis *in vitro* and *in vivo*. Moreover, it could reduce the migration, invasion and EMT. Mechanistically, sevoflurane could downregulate miR-26a expression and that miR-26a could negatively modulate FOXO1 activity. Thus, sevoflurane could increase FOXO1 activity. In the clinical samples, miR-26a expression was significantly upregulated, but FOXO1 was remarkably down-regulated and miR-26a expression in LSCC was linked with better prognosis. In conclusion, MiR-26a is increased and FOXO1 is reduced in human LSCC, Sevoflurane inhibits proliferation and mediates apoptosis of LSCC cells. Further, MiR-26a binds FOXO1 directly, and FOXO1 expression is down-regulated by Sevoflurane. Finally, Sevoflurane triggers LSCC cells apoptosis in vivo. Sevoflurane use to target miR-26a/FOXO1 may be a novel alternative for LSCC therapy.

## 1. Introduction

According to recent reports, more than 98% of laryngeal malignancies are well-differentiated squamous cell carcinomas [1], and its mortality rate remains very high. Multiple risk factors for laryngeal squamous cell carcinoma (LSCC) exists, for example, human papillomavirus infection, gastroesophageal reflux disease, cigarette smoking, alcohol ingestion [2]. The current management options for LSCC include surgery, chemotherapy, radiotherapy and target therapy [3]. Although surgery remains as the cornerstone for LSCC treatment, the resection margin, clinical stage and comorbidity can influence the prognosis [4]. Despite multiple treatment strategies, the five-year overall survival percentage of LSCC was estimated to be 49%, and the five-year disease-free survival rate was at 58% [5]. Consequently, alternative treatment methods are necessary, to enhance survival and reduce recurrence of LSCC.

Increasing evidence have revealed that anesthetic methods applied during surgery have an impact on the survival and recurrence of cancers [6]. It was reported that during surgery in esophageal cancer patients, the use of intravenous anesthesia with propofol was superior to volatile anesthesia in terms of overall survival and recurrence [7]. Sevoflurane is widely used anesthetic agent, and many studies have revealed that it has antitumor effects. For example, the migratory and invasive ability of lung carcinoma cells can be inhibited by sevoflurane, as sevoflurane inhibits the p38 MAPK signaling pathway [8]. Additionally, the proliferation and EMT of breast cancer cells can be suppressed by sevoflurane [9,10]. However, the role of sevoflurane in LSCC has not been studied.

MicroRNAs (miRNAs) are defined as small non-coding RNA molecules consisting of 19–23 nucleotides [11]. Reports have indicated that, miRNAs are important for gene expression, modulation, and transduction of cell signaling [12]. MiRNAs play a role in various steps which are important for tumor progression, including cell proliferation, survival, specialization, apoptosis and migration [10,13,14]. Further, miRNAs have been reported to have a therapeutic ability toward LSCC. MiR-1, for instance, reduces proliferation, migration and invasion through a negative fibronectin 1 (FN1) regulation in Hep-2 cells [15]. MiR-129-5p, which is reduced in primary LSCC, has also been reported to have a significant effect on cell growth and migration and lead to the arrest of cell cycle via APC targeting and STAT3 modulation to initiate apoptosis in Hep-2 cell lines [16].

Further reports have indicated that miR-206 has a minimized expression in LSCC tissues and is negatively linked with the T-, N-, and Clinical stages progression. Inducing high miR-206 levels leads to severe suppression of proliferation and invasion and increased apoptosis [17]. Mir-26a/miR-26b, specifically has been described as tongue squamous cell carcinoma (TSCC) suppressor via PAK1 targeting in Cal27 and SCC4 cells [18]. However, the precise molecular mechanism of miR-26a and its role in in the progression of LSCC needs further study.

Human Forkhead box O (FOXO) genes, including FOXO-1, −3a, −4, and −6 are important PI3K/Akt signaling effectors and modulate several processes, for instance regulation of cell cycle, oxidative stress responses, cell differentiation and tumorigenesis [19]. Importantly, FOXO1 is additionally a downstream protein of PI3K/AKT pathway. Activated AKT leads to FOXO1 phosphorylation, which is then exported into the cytoplasm from the nucleus and broken down by proteasomes [20]. Further, FOXO1 has been shown to induce the arrest of G1 phase cell-cycle in renal cell carcinoma and glioma cells due to the suppression of tumor regulator phosphatase and tensin homolog deleted on chromosome ten (PTEN), through the p27 upregulation [21].

The present study hypothesized the essential role of sevoflurane in the suppression of LSCC via miR-26a and FOXO1 targeting. This investigation aimed at determining the expression of MiR-26a and FOXO1 in human laryngeal squamous cell carcinoma, elucidating the effects of Sevoflurane in proliferation and apoptosis of LSCC cells. The study also aimed at establishing the role of Sevoflurane in LSCC cells apoptosis in vivo.

## 2. Materials and methods

### 2.1 Clinical samples

This research project was approved by the Ethical Committee of Huangshi Central Hospital of Edong Healthcare Group. A prior written informed consent was obtained from the study patients. The study enrolled 56 patients diagnosed with LSCC in Huangshi Central Hospital from December 2014 to December 2019. None of the patients under study had undergone radiotherapy, chemotherapy or any other specialized treatment prior to surgery. Diagnosis was independently confirmed by two senior pathologists. Samples were obtained from human tracheal epithelial cells (HTEpiC) of the patients under study and the tumor presence was confirmed by the senior pathologists. Clinical samples (n = 56) and normal adjacent choroidal tissues (n = 56) were stored at −80°C. The clinical and pathological features of patients are shown in Table 1.

**Table 1.** Clinicopathological characteristics of LSCC patients in this research

Groups	n/mean ± SD
Total	56
Gender	
Female	29 (51.8%)
Male	27 (48.2%)
Age	
≤ 60 years	26 (46.4%)
> 60 years	30 (53.6%)
pTNM	
T1	5 (8.9%)
T2	29 (51.8%)
T3	15 (26.8%)
T4	7 (12.5%)
Unknown	0
Lymph node metastasis	
No	38 (67.9%)
Yes	18 (32.1%)
Histopathological grade	
High	22 (39.2%)
Middle	24 (42.9%)
Low	8 (14.3%)
Unknown	2 (3.6%)

### 2.2 Cells and cell culture

Hep-2 and Tu177 cells (Human LSCC cell lines) were purchased from Chinese Academy of Biology, Shanghai, China. The cells were cultured in Dulbecco’s modified Eagle’s medium (Beyotime, China), supplemented with 10% fetal bovine serum (Gibco, USA). Hep-2 and Tu177 cells were incubated in a humid atmosphere (37°C and 5% CO_2_). The cells were passaged when cell confluence reached 70–80%. Cells were rinsed using PBS and digested using 0.25% trypsin. Lastly, the suspension of cells were centrifuged at 1000 rpm for 5 min and seeded into new plates.

### 2.3 LSCC cells treatment with sevoflurane

Initially, Hep-2 and Tu177 cells were inoculated onto 6-well plates. Sevoflurane was purchased from Sigma-Aldrich in liquid. It was diluted in cell culture medium by mixing for 30 min to produce relatively stable 10 mM solution. Then, LSCC cells were cultured in different concentration of sevoflurane for 6 h. The concentration of sevoflurane in culture medium were detected by a gas chromatography (GC model 6890 N; Agilent Technologies, Palo Alto, CA, USA) to ensure sevoflurane concentration in culture medium was stable.

### 2.4 Cell transfection

The following products were bought from Genomeditch (Shanghai, China): miR-26a mimic, mimic negative control (NC), miR-26a inhibitor and inhibitor negative control (NC). Lipofectamine 3000 (Invitrogen, USA) was employed as the transfection reagent and the transfection was performed based on manufacturer’s protocols. After an incubation of 48 h in 37°C and 5% CO2, the transfected cells were used for subsequent assays [22].

### 2.5 Quantitative real-time PCR (qRT-PCR)

Total RNAs from cells and tissues was extracted using Trizol reagent (Sigma-Aldrich, USA). Total miRNA was obtained using Molpure Cell/Tissue miRNA Kit (Yeasen, Shanghai, China). Additionally, mRNA was transcribed with Hifair III One Step RT-qPCR Probe Kit (Yeasen, Shanghai, China). At the same time, TaqMan MicroRNA Reverse Transcription Kit (Invitrogen, USA) was used to transcribe miRNA. SYBR green was obtained from Roche, Switzerland, and was used to perform real-time PCR. The primers in our experiments are listed in Table 2. U6 was used as the endogenous control for miR-195. The endogenous control for other primers was GAPDH. The 2^−∆∆Ct^ method was applied in determining the expressions of relative mRNA or miRNA [23].

**Table 2.** The primers used in this study

Gene	Primer 5ʹ→3’
MiR-26a	F: TGGCCTCGTTCAAGTAATCCAG
	R: GTCCCCGTGCAAGTAACCA
FOXO1	F: CAGCCGCCACATTCAACAGG
	R: GCTCTTGACCATCCACTCGT
GAPDH	F: GGAGCGAGATCCCTCCAAAAT
	R: GGCTGTTGTCATACTTCTCATGG
U6	F: CTCGCTTCGGCAGCACA
	R: AACGCTTCACGAATTTGCGT

### 2.6 Cell proliferation assay

CCK-8 kit (Beyotechnology, Shanghai, China) was used to assess cell proliferation. Approximately 4 × 10^4^ cell/ml, suspended in DMEM were inoculated in 96-well plates, and incubated for 12 h at 37°C and 5% CO_2_. Later, the cells were treated with 1, 2, 3, or 4 mM of Sevoflurane and incubated for 24 h. next,10 μL of CCK-8 solution was added in each well, incubated for 4 h and centrifuged for at 1750 x for 10 min. later, the supernatant was removed and 150 µL of DMSO was added in every well. The samples were then shaken for 10 min and OD values determined at 570 nm wavelength in spectrophotometer (Hitachi, Ltd., Tokyo, Japan). Each treatment was carried out in triplicates. The Inhibition rate was calculated as follows: Inhibition rate (%) = (control group A – experimental group A)/control group A x100% [24].

### 2.7 Wound healing assay

To assess the healing capability due to Sevoflurane treatment, wound healing assay was done as previously described [25]. About 4 × 10^5^ of Hep-2 and Tu177 cells per well were cultivated in a 6-well plate with DMEM augmented with 10% FBS to around 70% confluence. Wound was then made in the cell layer with a tip of a sterile pipette. The cells were then washed twice using PBS and cultured in the absence or presence of miR-26a inhibitor or sevoflurane, in 1% FBS for 48 h at 37°C. The cells were then observed microscopically, and the images were captured. The cell migration rates were assessed using the formula:

Migration rate = [width of (0–48 h)/width of 48 h] x 100%. The assays were independently done in triplicate.

### 2.8 Transwell invasion assay

To evaluate the invasion of Hep-2 and Tu177 cells, transwell invasion assay was utilized in this research [26]. Initially, Matrigel (Corning, USA) was placed on the top of the filter membrane of transwell insert and incubated at 37°C for about 30 min. Following that, 10 μl of cell suspension (1 x 10^6^ cells/ml) of transfected Hep-2 and Tu177 cells was added onto a transwell insert filter membrane (Millipore, USA) and incubated for 15 min to settle. 72 h later, the culture medium, transwell insert and Matrigel on the filter membrane were carefully removed. After that, 70% ethanol was added into each well and the transwell insert was immersed into the ethanol for fixation. What followed was that 0.2% crystal violet was added into each well to stain cells in the filter membrane. Finally, after crystal violet solution was removed with care, cells were visualized and images captured under phase-contrast microscope. Random observation of at least four fields was undertaken, and cells quantified using NIH-ImageJ software.

### 2.9 Flow cytometry

Apoptosis of tumor cells was determined using Pharmingen^TM^ PE Annexin V apoptosis detection kit (B.D., USA) and a flow cytometer (Beckman, USA). Summarily, about 1 × 10^6^ cells in 2 mL DMEM were seeded in 6-well plates (Corning, USA). The cells were then treated with sevoflurane. Cells were later incubated at 37°C for 12 h. P.E. Annexin V kit was utilized for assessment of apoptosis according to the manufacturer’s protocol. Data analysis was done using a flow cytometer.

### 2.10 Dual luciferase reporter assay

The predicted FOXO1 3′-UTR sequences containing the binding sites of miR-26a were amplified by PCR and then sub-cloned into a dual-luciferase reporter construct pMIR-GLO (Promega) forming the pMIR-GLO- FOXO1 -WT reporter vector. The mutant reporter vector pMIR-GLO- FOXO1 -MUT was made by a site-directed mutagenesis kit (New England Biolabs). Hep-2 and Tu177 cells were co-transfected with miR-26a mimics or scramble control and with pMIR-GLO- FOXO1-WT or MUT reporter vector through the use of Lipofectamine 2000 (Thermo Fisher Scientific) and incubated for 48 h. Luciferase activities were then determined using the Dual-Luciferase Reporter Assay System (Promega) [22]. All the assays were repeated thrice and plated in triplicates.

### 2.11 Immunohistochemistry

Immunohistochemistry assay was done using Dako Autostainer Link48 platform [27]. Summarily, endogenous peroxidase quenching was done with Flex Peroxidase Block (Dako) at a room temperature for 5 min. sections of TMA were incubated using rabbit polyclonal antibodies against E-cadherin (1/1000; Sigma Aldrich) and N-cadherin (1/1000; Sigma Aldrich) at room temperature. After an amplification with a rabbit linker (Dako) and two times rinsing in buffer, the slides were incubated using a horseradish peroxidase-labeled polymer coupled to secondary anti-mouse antibody for 20 min. Later, 3, 3ʹ-Diaminobenzidine substrate was applied for 10 min and Counterstained using Flex Hematoxylin (Dako). The slides were then washed for 5 min under tap water. Lastly, the slides were mounted using a coverslip post-dehydration.

## 2.12 protein extraction and Western blotting

Total and cellular proteins extraction was done using RIPA buffer (Beyotechnology, Shanghai, China) supplemented with protease inhibitor cocktail (Beyotechnology, Shanghai, China). Next, the cells were centrifuged for 20 min at 15,000 g and 4°C. The concentration of proteins was then assessed by Bradford assay and the obtained lysates loaded on Tris-HCl sodium dodecyl sulfate-polyacrylamide gel electrophoresis gels (SDS-PAGE) gel for electrophoresis and run at 100 V power. Later, the samples were transferred onto PVDF membranes (Thermo Fischer Scientific, USA) and blocked with 4% bovine serum albumin for 1 h at room temperature. The membrane was then incubated overnight with primary antibodies at 4 ^o^ C. Next, these membranes were incubated with the appropriate secondary antibodies at 24°C for approximately 1 h. Finally, protein bands would be visualized by ECL kits (Millipore, USA). The primary antibodies against E-cadherin (dilution: 1:1000, Abcam, UK; ab40772), N-cadherin (dilution: 1:1000, Abcam, UK; ab76001), vimentin (dilution: 1:1000, Abcam, UK; ab92547), FOXO1 (dilution: 1:1000, Abcam, UK; ab52857) and GAPDH (dilution: 1:1000, Beyotechnology, China; AF1186) were used in this research, and GAPDH was used as the internal control. Finally, bands were pictured using Immobilon^TM^ HRP substrate (Millipore) and analyzed using ImageJ software.

### 2.13 Animal experiments

The study was approved by the Huangshi Central Hospital of Edong Healthcare Group institutional Animal Care committee. Healthy 12 BALB/c nude male mice (weighing 18–20 g and 4–6 weeks old,) were bought from the animal center in our hospital, and kept in the animal rooms (10-h light/14-h dark cycle, 22–27°C and relative 40–60% humidity) under sterile environment with unlimited water and standard laboratory chow. These animals were randomly divided into 4 groups: (1) the control group (n = 3), (2) the sevoflurane treatment group (n = 5), (3) Sevoflurane treatment + antagomir negative control treatment group (n = 3) and (4) Sevoflurane treatment + miR-26a antagomir treatment group (n = 3). A pellet of 2.5 × 10^7^ Hep-2 cells were injected into the right flank of each BALB/c nude rat (18–20 g, 4–6 weeks old) subcutaneously. Seven days after feeding the mice, sevoflurane (50 mg/kg) was dissolved in 0.5 ml soybean oil and injected into mice intraperitoneally daily for 3 weeks. Mice were sacrificed via cervical dislocation, when tumor size reached 10 mm in diameter.

### 2.14 Statistical methods

Our data was analyzed on GraphPad Prism 8.0 and the results presentation was done in the form of mean ± standard deviation (SD). All our experiments were performed five times independently. Student’s t-test and one-way analysis of variance (ANOVA, Bonferroni post hoc test) were adopted in our analysis, depending on the experiment. Two-tailed P < 0.05 was set as the statistical threshold.

## 3. Results

### 3.1 MiR-26a is increased and FOXO1 is suppressed in human LSCC

**Figure 1.**
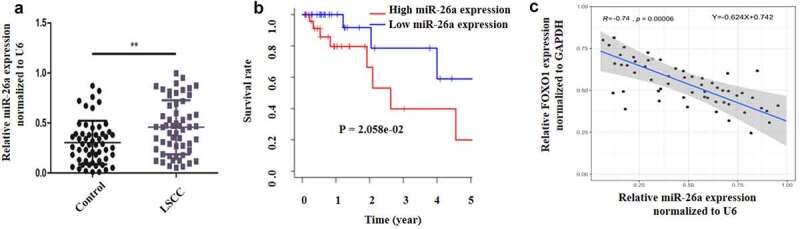
MiR-26a is increased and FOXO1 is suppressed in human LSCC. (a) MiR-26a expression in LSCC and normal adjacent tissues. (b) The survival rate curve in high miR-26a expression or low miR-26a expression. (c) The link between miR-26a and FOXO1 determined by Pearson correlation. *N* = 5; **P < 0.05 by two-sided Student’s t test, versus the control group

LSCC is a laryngeal malignancy with a high mortality rates, and its treatment remains difficult. Sevoflurane is a widely used anesthesia during surgery and has been proposed to have anti-tumor effect. The current investigation postulated that Sevoflurane induces apoptosis and anti-proliferative effects on LSCC through miR-26a/FOXO1. The study aimed at assessing whether Sevoflurane induces apoptosis of LSCC and the involvement of miR-26a/FOXO1 axis. First, to determine the expression of miR-26a and FOXO1 in human LSCC, 56 LSCC clinical samples and the adjacent normal skin tissues were obtained and examined by RT-qPCR. As per the results, miR-26a expression was upregulated in LSCC samples in comparison to the control samples (Figure 1a). However, FOXO1 expression was significantly suppressed in the LSCC positive clinical samples compared to the control samples (Figure 1b). Additionally, miR-26a expression was negatively linked to FOXO1 expression (Figure 1c). Together, these data indicated that MiR-26a is upregulated and FOXO1 is down-regulated in human LSCC.

### 3.2 Sevoflurane induces apoptosis, reduces metastasis and affects EMT in LSCC

**Figure 2.**
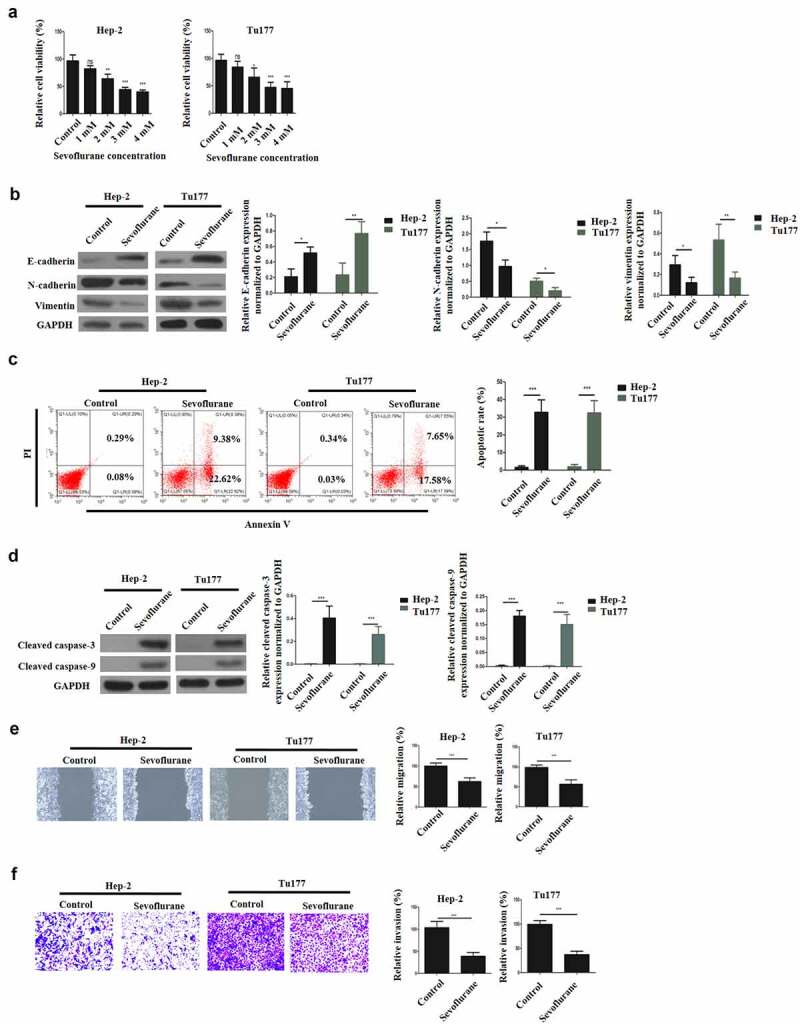
Sevoflurane induces apoptosis, reduces metastasis and affects EMT in LSCC. (a) Cell viability assessment by CCK-8. Hep-2 and Tu177 cells were treated with either control or Sevoflurane (1 mM, 2, mM, 3 mM or 4 mM) and cell proliferation was determined. (b) Western blot assays were carried out to determine E-cadherin, N-cadherin and vimentin expressions following the treatment of tumor cells with 4 mM of Sevoflurane. (c) Flow cytometry technique was used to examine apoptosis following treatment of LSCC cells with 4 mM of Sevoflurane. (d) Western blots assays were used to determine the apoptosis proteins cleaved caspase-3 and cleaved caspase-9 expression. (e) Wound healing assay was carried out to assess the migration of Hep-2 and Tu 177 cells following treatments with 4 mM of Sevoflurane. (f) Transwell technique was used to determine invasion in LSCC cells following Sevoflurane treatments. *N* = 5; *P < 0.05, **P < 0.01, ***P < 0.001 by two-sided Student’s t test, versus the control group

To elucidate the effects of Sevoflurane on LSCC, various concentration of sevoflurane (1, 2, 3, and 4 mM) were first used to treat LSCC for 4 h and the effects on cell viability was examined through CCK-8 assay. According to the results, treatment with sevoflurane reduced cell viability in a dose-dependent manner, with the least proliferation reported after treatment with the highest Sevoflurane dosage (Figure 2a). Consequently, 4 mM sevoflurane was chosen as the most effective concentration for our subsequent experiments. Next, western blot assays were performed to study the effects of Sevoflurane on EMT. According to the results, E-cadherin expression was significantly upregulated in the sevoflurane-treated cells compared to the controls. However, the expression of vimentin and N-cadherin expressions were significantly down-regulated in the sevoflurane-treated cells as compared to the control groups (Figure 2b).

To study the effects of Sevoflurane treatment on apoptosis, apoptotic cells expression was studied by Annexin V and flow cytometry. The results indicated a significantly increased apoptotic cells in both Hep-2 and Tu177 cells following the treatment with sevoflurane, as compared to the non-treated cells (Figure 2 C). Further investigation of apoptosis was undertaken through western blot to analyze the expression of caspace 3 and caspase 9 proteins expression. The observations demonstrated a remarkably elevated caspase 3 and caspase 9 proteins expression in the treated cells than in the control group, as shown in Figure 2d. Next, wound healing assays were performed to assess cellular migration. As is illustrated in Figure 2e, the migration of LSCC cells was significantly reduced by sevoflurane treatment, in comparison to the non-treated group. Finally, the effect of treatment on invasion was determined using transwell assays. The observations confirmed that, sevoflurane treatment significantly decreased cell invasive ability, in comparison to the non-treatment group (figure 2f). Together, these data confirmed that sevoflurane could inhibit the malignant progression of LSCC.

### 3.3 Sevoflurane mediates its effects on LSCC via miR-26a targeting

**Figure 3.**
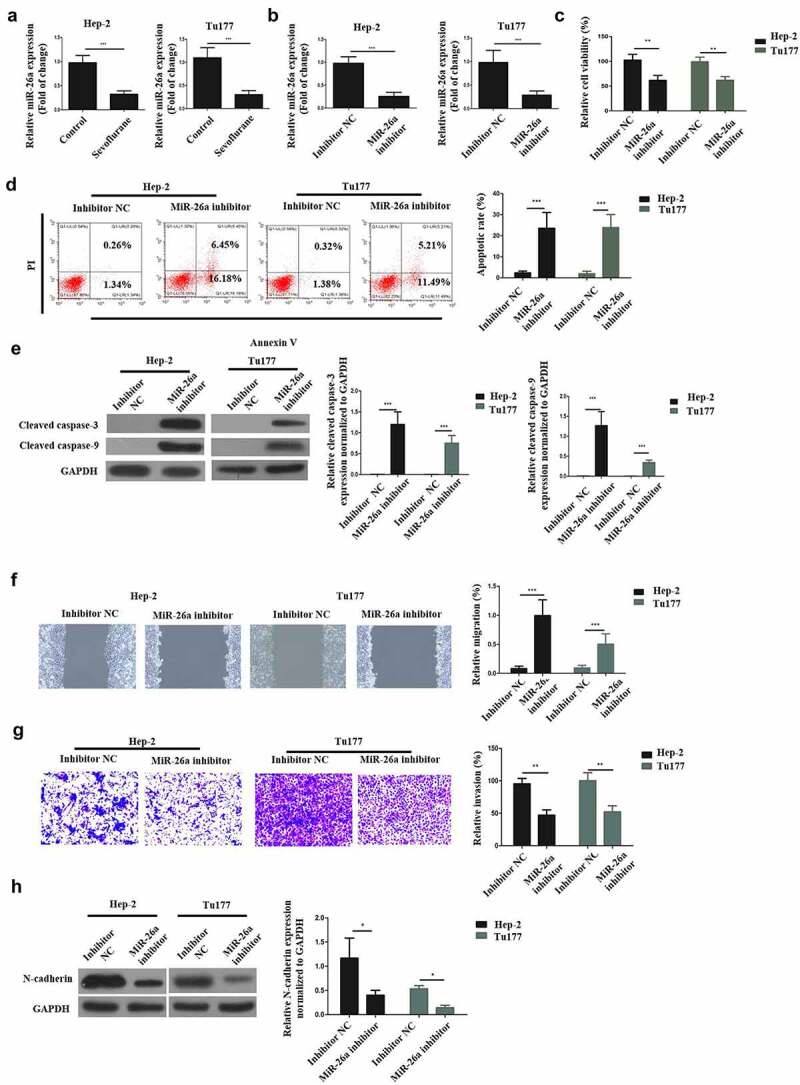
Sevoflurane mediates its effects on LSCC via miR-26a targeting. (a) RT-qPCR was used for the assessment of MiR-26a expression following treatment of Hep-2 and Tu177 cells with 4 mM of Sevoflurane, or control. (b) RT-qPCR assay assessing the MiR-26a expression following the treatment of LSCC cell lines with miR-26a-inhibitors or its negative controls. (c) Cell viability assessment by CCK-8 following the transfection of Hep-2 and Tu 177 cells with miR-26a inhibitors or their NC. (d) Flow cytometry assay to examine apoptosis following transfection of LSCC cell lines with miR-26a-inhibitors or their negative controls. (e) Western blots for assessment of cleaved caspase-3 and cleaved caspase-9 apoptotic proteins expressions following transfection of cells with miR-26a inhibitors or their controls. (f) Wound healing assay to study the migration of tumor cells following transfections with miR-26a inhibitors or their controls. (g) Transwell assays was used for determining invasion in LSCC cell lines following the transfection with miR-26a inhibitors. (h) Western blots assays for the assessment of E-cadherin, N-cadherin and vimentin expressions in cells transfected with miR-26a-inhibitors or their controls. *N* = 5; *P < 0.05, **P < 0.01, ***P < 0.001 by two-sided Student’s t test, versus the control group

RT-qPCR was used to understand the relationship between Sevoflurane and miR-26a in the initiation of LSCC anti-proliferative effects. According to the findings, sevoflurane significantly reduced miR-26a expression, compared to the control non-treated groups in both LSCC cell lines (Figure 3a). Next, Hep-2 and Tu177 cells were transfected with miR-26a inhibitor and inhibitor negative control (NC) respectively. The transfection success was then determined by assessing the miR-261 mRNA expression through RT-qPCR. According to the observations, miR-26a expression was significantly reduced following the transfection of both cells with miR-26a inhibitor, as compared with transfection with the inhibitor NC (Figure 3b).

Next, cell viability was determined after transfection with the inhibitors or the NC, through CCK-8 assays. The observations demonstrated a significantly reduced cell viabilities in both the cells following transfection with the miR-26a inhibitors, as compared with the inhibitor-NC, as shown in Figure 3c. Next, flow cytometry and Western blot assays were used to determine apoptosis following transfection with miR-26a-inhibitors. The observations indicated that the apoptosis of LSCC cells is significantly enhanced by the transfection of miR-26a inhibitor as compared to transfection with inhibitor-NC (Figures 3d and 3e). Further, the effects of miR-26a transfection on wound healing was investigated through transwell assays.

The observations confirmed that following miR-26a inhibitor transfection, the migration and invasion of LSCC cells was significantly decreased as compared to the inhibitor-NC transfection (figures 3f and 3g). Finally, western blot experiments were used to determine the N-cadherin expression after miR-26a inhibitor transfection. The results demonstrated a significantly down-regulated N-cadherin expression after miR-26a transfection in both the Hep-2 and Tu177 cells, as compared to the transfection with inhibitor-NC (Figure 3h), indicating that miR-26a could decrease the EMT of LSCC. Overall, these observations confirm that Sevoflurane initiates LSCC anti-proliferative effects via miR-26a targeting.

## 3.4 miR-26a binds FOXO1 directly

**Figure 4.**
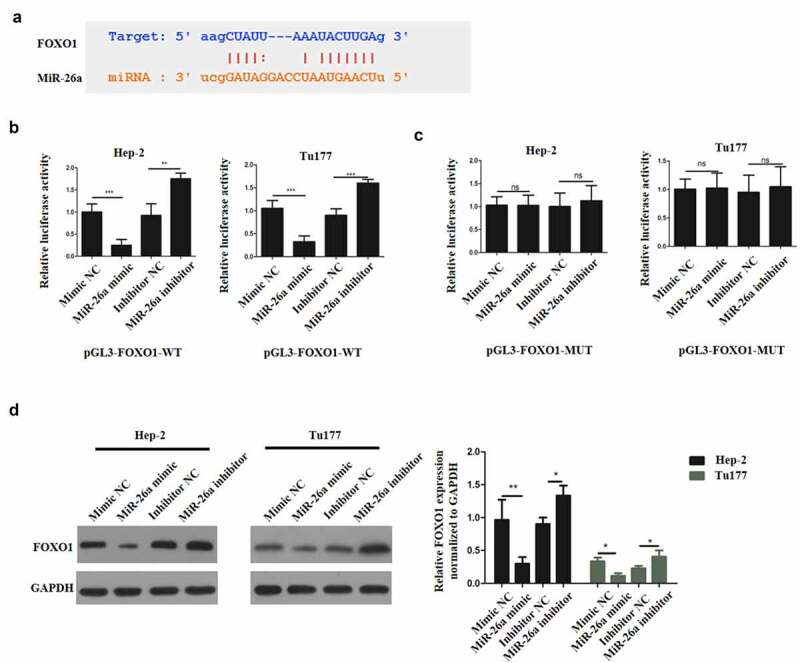
MiR-26a binds FOXO1 directly. (a)The complementary sequence between miR-26a and FOXO1. (b) Luciferase activity in wild type luciferase constructs. (c) Luciferase activity in mutant-type luciferase constructs. (d) FOXO1 expression following miR-26a inhibitor transfection. *N* = 5; ***P < 0.001 by two-sided Student’s t test, versus the control group

To identify the miR-26a target and elucidate the mechanisms underlying its functions in LSCC, Starbase (http://starbase.sysu.edu.cn/index.php) was used. The prediction results demonstrated the existence of a complementary sequence between miR-26a and FOXO1 (Figure 4a). Next, dual luciferase reporter assay was carried out on two LSCC cell lines to determine if miR-26a directly binds to the FOXO1 3ʹUTR. Hep-2 and Tu177 cells were transfected with pGL3-FOXO1-WT luciferase construct and miR-26a mimic, mimic negative (NC), miR-26a inhibitor or inhibitor negative control (NC) respectively. The co-transfection of pGL3-FOXO1-WT luciferase construct and miR-26a mimic downregulated the luciferase activity, whereas the co-transfection of pGL3-FOXO1-WT luciferase construct and miR-26a inhibitor increased the luciferase activity (Figure 4b).

In the meantime, cells were transfected with pGL3-FOXO1-MUT luciferase construct and miR-26a mimic, mimic negative control (NC), miR-26a inhibitor or inhibitor negative control (NC) respectively. The co-transfection of pGL3-FOXO1-MUT luciferase construct and miR-26a mimic or miR-26a inhibitor did not influence the luciferase activity (Figure 4c). Moreover, when Hep-2 and Tu177 cells were transfected with mimic NC, miR-26a mimic, inhibitor NC and miR-26a inhibitor respectively, we found that FOXO1 expression was significantly reduced by miR-26a mimic, but it was significantly upregulated by miR-26a inhibitor in both Hep-2 and Tu177 cells (Figure 4d). Therefore, these observations confirmed that miR-26a could target FOXO1 directly to mediate the effects of sevoflurane on LSCC.

### 3.5 *Sevoflurane inhibits EMT of LSCC and promotes its apoptosis* in vivo

**Figure 5.**
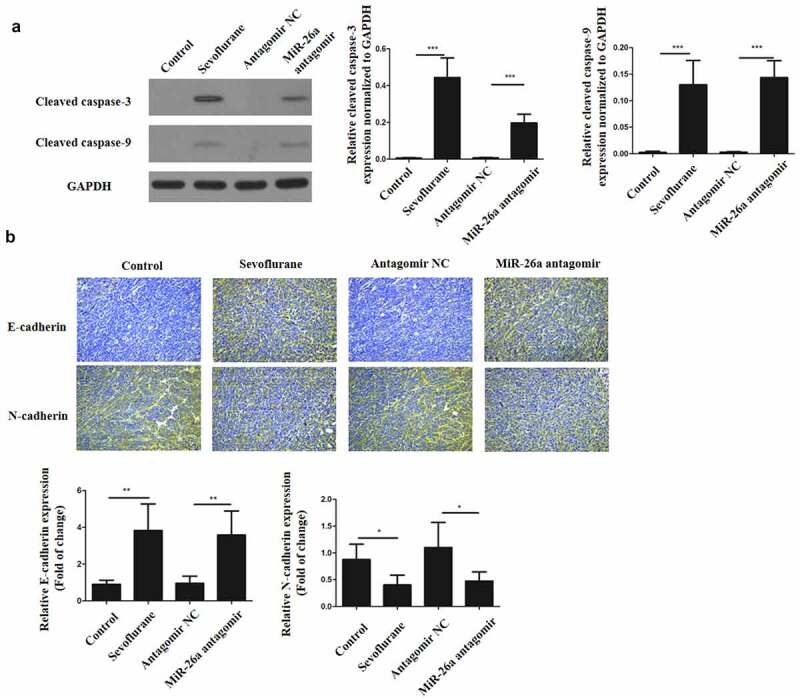
Sevoflurane inhibits EMT of LSCC and promotes its apoptosis *in vivo*. (a) Western blots to determine the expressions of cleaved caspase-3 and cleaved caspase-9 following transfection of LSCC cells with sevoflurane, miR-26a-antagomir or their respective negative controls. (b) Immunohistochemistry staining for N-cadherin and E-cadherin. *N* = 5; *P < 0.05 by two-sided Student’s t test, versus the control group

To verify our findings *in vitro*, Hep-2 and Tu177 cells were injected subcutaneously into nude mice to initiate tumors. The mice were then treated with 4 mM of Sevoflurane and apoptosis determined in tissues proteins through western blots. As shown in Figure 5a, cleaved caspase-3 and cleaved caspase-9 in sevoflurane treatment group and miR-26a antagomir treatment group were significantly increased in comparison to the control groups. In addition, immunohistochemistry staining for E-cadherin and N-cadherin was performed on these tumors. E-cadherin expression was significantly increased in sevoflurane treatment group and miR-26a antagomir treatment group, as compared to the control groups. However, N-cadherin expression in both sevoflurane treatment and miR-26a antagomir treatment group was downregulated as compared to the control groups (Figure 5b). Thus, these observations confirm that sevoflurane has anticancer effects *in vivo*.

## 4. Discussion

LSCC has a high recurrence rate and a high metastasis chance. The conventional treatment methods for LSCC include surgery and chemotherapy which have had long time issue of remission. In addition, several drugs pose a dangerous side effect. As a result, developing more effective novel LSCC treatments alternative is necessary. The current research investigated the effects of Sevoflurane in LSCC treatment. The findings confirmed that Sevoflurane could promote the apoptosis of LSCC and down-regulate the proliferation in LSCC both in vitro and in vivo.

According to various studies, different anesthetic techniques can influence the outcomes of cancer patients who have received surgery [6,28]. Severolurane has been proven to have anti-neoplastic effects on multiple carcinomas, for instance, breast cancer, colorectal cancer and lung cancer [24]. In agreement with these previous reports, the current research notes that sevoflurane could induce the apoptosis in LSCC both *in vitro* and *in vivo* and reduce their viability, migration, invasion as well as their EMT. Increasing the apoptosis of LSCC remains a major strategy against cancer [29]. The activity of cleaved caspase-3 and cleaved caspase-9 was also upregulated by sevoflurane and flow cytometry results also confirmed the pro-apoptotic effects of sevoflurane. Generally, cancer migration and invasion are important traits for dissemination and neo-angiogenesis [30]. Epithelial-mesenchymal transition is a process where the phenotypes of epithelial cancer cells change into more invasive and metastatic phenotypes [31]. The EMT process makes cancer cells resistance cells to drugs [32]. According to the observed anticancer properties of sevoflurane in this experiment, sevoflurane can be considered as an antitumor anesthetic agent for LSCC.

Moreover, sevoflurane downregulates miR-26a expression, consequently leading to induction of apoptosis and the suppression of migration, invasion and EMT in LSCC. A study found that sevoflurane could influence the expression of miR-26a in rat’s lungs [33]. Thus, our finding was consistent with other research discoveries. MiR-26a role in cancer has been elucidated and it has been described as an anti-tumor molecule. For instance, miR-26a can suppress the proliferative ability and metastasis of gastric carcinoma by modulating FGF9 [34]. In addition, the migration and growth of breast carcinoma cells could be suppressed by miR-26a, and miR-26a can even suppress EMT [35]. Mechanisms of Invasion and metastasis is closely linked to EMT. Being a critical EMT molecule, E-cadherin has a role in maintaining the normal intercellular connections’ stability, and its expression levels negatively correlates to EMT occurrence and tumor invasion capability [32]. The process of EMT is characterized by down-regulated E-cadherin expression but elevated N-cadherin levels.

Mechanistically, it was discovered that miR-26a could bind FOXO1 and downregulate its activity. This indicates that sevoflurane could increase the expression of FOXO1. In many carcinomas, FOXO1 is recognized as a tumor suppressor. FOXO1, which is a downstream effector of PI3K/AKT signaling, serves to regulate cell differentiation and apoptosis [36]. This gene can decrease the migration and invasion of prostate carcinoma cells by interacting with Runx2 [37]. In addition, it can modulate cell apoptosis through CDK2 [38]. It is noteworthy that the invasion, EMT and metastasis of hepatocellular carcinoma can be decreased by FOXO1 via ZEB2 [39]. Thus, sevoflurane is an anticancer agent by upregulating FOXO1 activity.

## 5. Conclusion

MiR-26a is increased and FOXO1 is reduced in human laryngeal squamous cell carcinoma, Sevoflurane inhibits proliferation and mediates apoptosis of LSCC cells. Further, MiR-26a binds FOXO1 directly, and FOXO1 expression is down-regulated by Sevoflurane. Finally, Sevoflurane triggers LSCC cells apoptosis in vivo. The use of Sevoflurane to target miR-26a/FOXO1 may be a novel and effective alternative for laryngeal squamous cell carcinoma therapy.
